# Pack Cementation Route to Ag_2_Se: Correlating Structure, Phase Formation, and Thermoelectric Performance

**DOI:** 10.3390/nano15211676

**Published:** 2025-11-04

**Authors:** Aikaterini Teknetzi, Dimitrios Stathokostopoulos, Savvas Hadjipanteli, Isaak Vasileiadis, Evangelia Tarani, Nikolaos Hastas, Eleni Pavlidou, Thomas Kehagias, Theodora Kyratsi, George Vourlias

**Affiliations:** 1 School of Physics, Faculty of Sciences, Aristotle University of Thessaloniki, 54124 Thessaloniki, Greece; dstat@physics.auth.gr (D.S.); isvasile@physics.auth.gr (I.V.); etarani@physics.auth.gr (E.T.); nhastas@auth.gr (N.H.); elpavlid@auth.gr (E.P.); kehagias@auth.gr (T.K.); 2 Department of Mechanical and Manufacturing Engineering, University of Cyprus, Nicosia 1678, Cyprus; hadjipanteli.savvas@ucy.ac.cy (S.H.); kyratsi.theodora@ucy.ac.cy (T.K.)

**Keywords:** silver selenide, low-temperature thermoelectrics, pack cementation, microstructure, phase transition, chemical state, Seebeck coefficient, thermal conductivity

## Abstract

Silver selenide (Ag_2_Se) is a promising thermoelectric material for near-room-temperature applications, yet its scalable fabrication remains challenging due to limitations in conventional synthesis routes and the strong dependence of its properties on processing conditions. In this work, the pack cementation technique is introduced as a novel cost-effective, and industrially viable route for producing *β*-Ag_2_Se powders. The influence of synthesis parameters on phase formation, composition, and microstructure is examined, and their correlation with thermoelectric behavior is studied to establish clear structure–property relationships. The resulting Ag_2_Se is comprehensively evaluated for quality and performance. Phase-pure orthorhombic *β*-Ag_2_Se with near-stoichiometric composition and a uniform microstructure was successfully synthesized, with phase purity preserved after consolidation without secondary phases. The material exhibited competitive thermoelectric performance, achieving a maximum *ZT* = 0.63 at 352 K and stable operation up to ~375 K. These findings demonstrate that pack cementation can deliver high-quality Ag_2_Se with competitive efficiency, highlighting its potential for future optimization and large-scale production.

## 1. Introduction

Thermoelectric materials, which directly convert heat into electricity and vice versa, have attracted significant attention over the last decades as a sustainable means of exploiting waste heat from power plants, vehicles, buildings, electronic devices or even human body. The efficiency of a thermoelectric material is described by the dimensionless figure of merit, *ZT*, defined as $ZT=\sigma {\;S}^{2}\;T/\kappa$, where *σ* is the electrical conductivity, *S* the Seebeck coefficient, *κ* the total thermal conductivity and *T* the absolute temperature. Practical conversion efficiency is achieved when *ZT* values exceed 1 in the temperature region of interest. In the ongoing search for new candidates with improved *ZT* values that would enable the construction of more cost-effective thermoelectric devices, new material families have entered the spotlight, including filled skutterudites, metal chalcogenides, clathrates, half-Heusler alloys, nanocomposites, silicides organic materials etc. [1,2].

Among these, silver selenide (Ag_2_Se) stands out as one of the most notable transition-metal chalcogenides for near room-temperature thermoelectric applications [3,4]. Its phonon-glass electron-crystal (PGEC) structure imparts unique properties, including remarkably reduced phonon propagation and superior carrier mobility [5]. This compound undergoes a first order phase transformation at approximately 407 K, shifting from the orthorhombic semiconducting *β*-phase to the cubic superionic *α*-phase [6,7]. In the superionic state, the Ag ions become mobile, moving easily through the rigid Se sublattice. However, the maximum *ZT* values are expected around room temperature where *β*-Ag_2_Se phase is stable. This low-temperature phase is an n-type semiconductor with a narrow bandgap (~0.04–0.2 eV [8,9,10]), high electron mobility, low electron effective mass, and weak chemical bonding, similar to the superionic phase. These special features yield a high electrical conductivity (*σ* ≈ 2000 S cm^−1^), a moderately large Seebeck coefficient (*S* ≈ −150 μV K^−1^), and intrinsically low lattice thermal conductivity (*κ*_L_ ≈ 0.2–0.5 W m^−1^ K^−1^) at 300 K [8,10,11,12]. High *ZT* values of 0.8–1.2 have been reported in the range of 300–400 K [6,13,14,15,16,17] making *β*-Ag_2_Se a promising candidate for low-temperature thermoelectric applications, such as local cooling of microelectronics in communication systems or energy harvesting from the low-grade waste heat below 400 K.

Interestingly, there is a significant discrepancy in the reported thermoelectric performance of Ag_2_Se prepared by different approaches, arising from the challenges associated with its synthesis and processing [6,9,13]. The low solubility of excess Ag or Se, along with the difficulty in precisely controlling stoichiometry, can induce compositional and microstructural inconsistencies [13]. Consequently, the strong dependence of the electrical and thermal transport properties on microstructure, homogeneity, and stoichiometry leads to constraints regarding the successful synthesis of high-quality *β*-Ag_2_Se with low carrier concentration. Numerous methods have been attempted to obtain silver selenide. Techniques based on solid-state reactions are a common approach, including annealing at high temperatures, mechanical alloying via high-energy ball milling, room-temperature grinding, and pulsed hybrid reactive magnetron sputtering [6,10,11,13,16,18]. Silver selenide thin films are typically prepared by evaporation methods [19]. Traditional melt-growth techniques have also been employed, with bulk Ag_2_Se polycrystals grown via zone-melting [9], and Ag_2_Se powders synthesized using melting-annealing methods, usually followed by sintering techniques (hot press or spark plasma sintering) [13,14,20]. Additionally, chemical methods for synthesizing nanostructured Ag_2_Se have been proposed, including electrochemical deposition, hydrothermal preparation, and other wet chemistry processes [21,22,23,24,25].

However, the high temperatures required by melting methods can result in considerable inhomogeneity in the material, since the high mobility of Ag^+^ ions in the superionic *α*-Ag_2_Se phase hinders the controlled distribution of silver. Furthermore, the highly volatile nature of Se complicates the formation of stoichiometric Ag_2_Se at temperatures close to its boiling point (958 K) [6]. Selenium loss may also occur during post-treatment at elevated temperatures (e.g., annealing or sintering). The elevated synthesis temperatures and prolonged annealing times in melting processes and certain solid-state reactions lead to excessive energy and time consumption. On the other hand, mechanical alloying methods often suffer from oxidation, significant agglomeration of the components, and contamination from the milling tools, affecting compositional control. Wet chemistry methods are prone to the formation of secondary phases and generally offer a relatively low yield [6,23]. Finally, the specialized equipment and/or experimental conditions required for some techniques, such as pulsed hybrid reactive magnetron sputtering and high-vacuum evaporation methods, prevent their large-scale application. All these factors limit the potential for scaling up Ag_2_Se production to industrially relevant levels.

The development of a preparation procedure that addresses these issues while enabling industrial-scale production of Ag_2_Se remains an open field, motivating the exploration of routes capable of producing material of good quality and performance through processes feasible for large-scale implementation. The pack cementation technique offers advantageous features that have established it in industrial applications, particularly for the deposition of anti-corrosion and wear-resistant coatings on various substrates [26,27]. It is a simple and low-cost process that does not require specialized equipment. The energy consumption is lower than that of other methods (e.g., melting techniques), as the desired phases can form at lower temperatures and within shorter timeframes via gas phase reactions, vapor transfer, nucleation and crystal growth. Furthermore, it is a scalable and highly reproducible process that meets the demands of industrial-scale production. The absence of any toxic by-products released into the environment during synthesis further enhances the ecological appeal of this method. Pack cementation can also be applied to prepare powder products, but its potential in this area has not been extensively explored yet. We successfully employed pack cementation to prepare high-quality, high-performance thermoelectric silicides in powder form, including Mg_2_Si, CrSi_2_, and MnSi_1.75_ both pristine and doped [28,29,30,31].

Given these advantages, pack cementation was selected as a promising route for Ag_2_Se production. In this study, we present a comprehensive study on the synthesis and characterization of β-Ag_2_Se powders produced by the pack cementation method. Particular emphasis is placed on the role of synthesis time in tailoring the final material, providing insights into its influence on phase formation, grain morphology, and microstructural evolution. The resulting samples are systematically examined with respect to their chemical composition, crystal structure, and microstructural features, allowing us to establish a direct correlation between processing conditions and structural quality. In addition, the intrinsic thermal phase transition of *β*-Ag_2_Se is examined in order to determine its stability across different temperature regimes. Finally, these structural, compositional, and thermal characteristics are linked to the measured thermoelectric properties, enabling a critical evaluation of how microstructure and phase purity impact its thermoelectric performance.

## 2. Materials and Methods

For the synthesis of Ag_2_Se samples, Ag (99.9%, −325 mesh) and Se (99.5%, −325 mesh) powders, both supplied by Thermo Scientific (Waltham, MA, USA), were weighed in an atomic ratio of 2:1 and mixed with 3 wt.% NH_4_F, which serves as the activator. The pack powder mixtures were placed in alumina crucibles and subsequently sealed in specialized metal crucibles, both bearing holes to facilitate the removal of trapped oxygen. The pack mixtures were then heated at 240 °C in a tube furnace for 3 h to 4 h and afterwards they were left to cool down to room temperature. Prior to heating, the system was purged with a rotary pump, and the whole synthesis and cooling process took place under argon flow (Ar 99.998%) to prevent oxidation. Typically, the pack cementation process can be described as follows: upon heating the powder mixture containing the constituent elements of the desired compound and a halide activator, the activator decomposes and predominantly reacts with one of the elements, the metal donor. Vapors of metal halide species are produced and transferred to the surface of the other elemental powder, which plays the role of the base material. There, the gaseous products decompose and deposit the donor in a more reactive form. The latter reacts with the base element, initiating the solid-state diffusion stage, facilitated by the heating. The diffusion process results in phase formation between the two elements. The chemical reactions are recycled through the halide by-products produced during the donor deposition, and the whole process is completed once the donor is totally consumed. The final product is the desired compound in powder form.

The synthesized Ag_2_Se powders were characterized with respect to their structure and phase composition using X-ray diffraction (XRD) analysis with a two-cycle Rigaku Ultima^+^ powder X-ray diffractometer, employing CuKa radiation (40 kV/30 mA). The morphology and the chemical composition of the samples were studied by a JEOL JSM-7610F Plus scanning electron microscope (SEM), equipped with an Oxford AZTEC ENERGY ADVANCED X-act energy dispersive X-ray spectroscopy (EDS) analyzer. Additionally, the chemical environment of their constituent elements was further investigated by X-ray photoelectron spectroscopy (XPS), employing a KRATOS Analytical AXIS Ultra^DLD^ system with Al monochromatic X-ray source (λ_Ka_ = 1486.6 eV). The high-resolution XPS spectra were calibrated with respect to the C-C 1s peak at 284.6 ± 0.2 eV, which originates from atmospheric contamination. The background was fitted with Shirley (non-linear) baselines. The experimental curves were fitted using a combination of Gaussian (70%) and Lorentzian (30%) distributions, except for metallic contributions, which were fitted with an asymmetric peak shape. The grain morphology, microstructural features, and phase composition of selected samples were investigated using transmission electron microscopy (TEM) techniques. All TEM analyses were performed on a JEOL JEM-F200 TEM/STEM CFEG microscope, providing a point resolution of 0.19 nm in TEM mode and 0.14 nm in scanning TEM (STEM) mode, operated at 200 kV. For chemical analysis and elemental mapping, the microscope is coupled with an Oxford Aztec X-Max EDS detector, enabling simultaneous structural and compositional characterization. The phase transition of Ag_2_Se was examined with a NETZSCH Polyma 214 differential scanning calorimeter (DSC), calibrated with indium and zinc standards. The heating-cooling cycle was recorded at a scan rate of 10 K/min, using 10 mg of powder sealed in aluminum pans.

The powders were hot-pressed into pellets at 600 °C applying 80 MPa for 1 h and under nitrogen flow. The density of the pellets was calculated by the geometrical method based on their measured mass and volume, and the relative densities were greater than 97% of the theoretical value. Hall effect measurements were conducted on the optimal specimen in the van der Pauw configuration, using a custom-made apparatus. The room-temperature data were collected under a magnetic field of ± 0.5 T and an alternating electric current of 15 mA. In terms of thermoelectric characterization, the Seebeck coefficient and electrical conductivity were measured utilizing a commercial ZEM-3 ULVAC–RIKO apparatus, while the thermal diffusivity (*D*) measurements were conducted with a Netzsch LFA 457 laser flash apparatus. Thermal conductivity, *κ*, was subsequently calculated through the relation *κ* = *D* ρ *C_p_*, where ρ presents the pellet density and *C_p_* is the specific heat capacity. The uncertainty for the electrical conductivity, Seebeck coefficient and thermal conductivity was 5%, leading to a combined uncertainty of about 10% for the power factor and 15% for the *ZT*.

All synthesis, processing, and characterization equipment is located at Aristotle University of Thessaloniki, except for the ZEM-3 ULVAC–RIKO and Netzsch LFA 457 apparatus, which are located at the University of Cyprus.

## 3. Results and Discussion

### 3.1. Phase Composition, Structure, and Chemical Analysis

**Figure 1:**
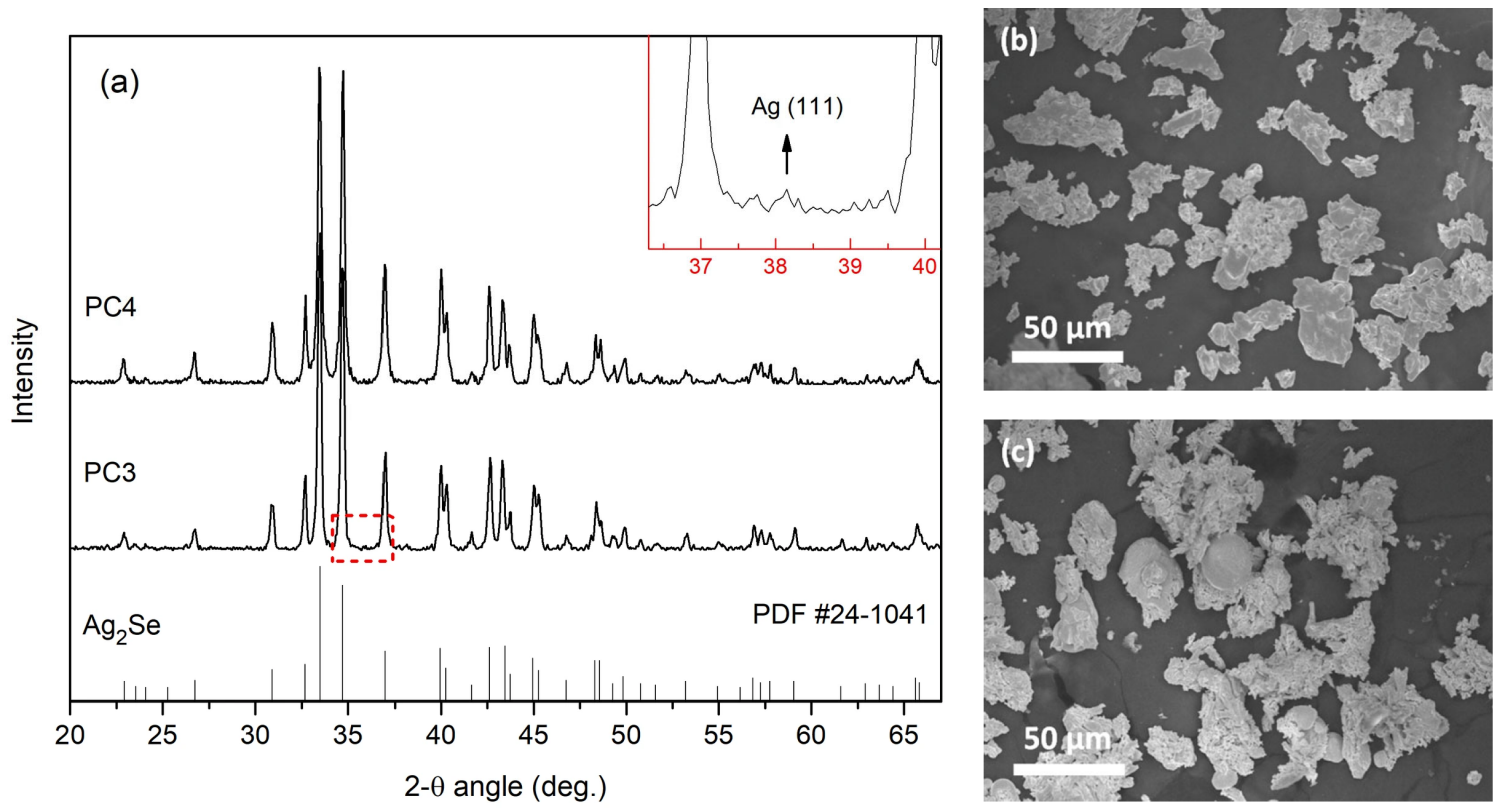
X-ray diffraction patterns of powders PC3 and PC4 (**a**). The material is identified as *β*-Ag_2_Se in both cases, although a weak peak of Ag trace may also be present for PC3 (inset). SEM micrographs of powders PC3 (**b**) and PC4 (**c**), consisting of irregularly shaped particles of several microns that tend to aggregate.

The powders synthesized after 3 h and 4 h of heating are hereafter referred to as PC3 and PC4, respectively. The formation of Ag_2_Se from elemental Ag and Se is known to occur through Se volatilization and the direct reaction of solid Ag with these vapors, a process that can take place even at ambient conditions, although the reaction rate depends on several factors. Yang et al. [6] reported that mechanical stirring and hand grinding can accelerate the reaction, but are not essential for the reaction to happen. We speculate that at least two mechanisms may contribute to Ag_2_Se synthesis during pack cementation: (a) spontaneous dissociative adsorption of Se vapor by Ag, and (b) thermochemical reactions involving NH_4_F decomposition products, which may accelerate the reaction rate. Further investigations are ongoing to clarify the exact mechanisms involved. Figure 1a presents the X-ray diffraction patterns of PC3 and PC4, where all the peaks are identified as the orthorhombic *β*-Ag_2_Se phase with space group P2_1_2_1_2_1_ (indexed by JCPDS #24-1041) [32]. Nonetheless, a closer inspection of the PC3 pattern reveals a weak peak at 2θ = 38.15°, as shown in the inset of Figure 1a, which may be indexed as the (111) reflection of pure Ag (JCPDS #65-2870) [32], indicating the presence of Ag clusters in the material. Since the peak is small and not well defined, further confirmation using a complementary characterization technique is necessary. The excess unreacted Ag could, in principle, arise from either incomplete synthesis or from Se loss due to evaporation. However, the absence of a corresponding Ag peak in the pattern of PC4 indicates that incomplete synthesis is the main contributor, while any Se loss that may occur in both samples is likely negligible. These observations suggest that the longer heating time n PC4 has likely promoted the completion of the synthesis process. No substantial difference in the morphology of the two powders is observed, as can be seen in the representative SEM micrographs in Figure 1b,c. Both powders consist of irregularly shaped particles, each measuring several microns in size. Many of these particles tend to agglomerate, forming larger aggregates of up to approximately 50 μm. The actual elemental composition of the powders, averaged from 25 EDS point measurements taken at random spots, was determined to be about Ag_1.94_Se for PC3 and Ag_2.03_Se for PC4. The measured stoichiometry is in close agreement with that of Ag_2_Se, consistent with the *β*-Ag_2_Se phase identified in the XRD patterns. Nonetheless, some regions exhibiting an excess of Ag (≥75 at.%) were also detected in powder PC3. These regions probably contain unreacted Ag, further corroborating the XRD findings.

**Figure 2:**
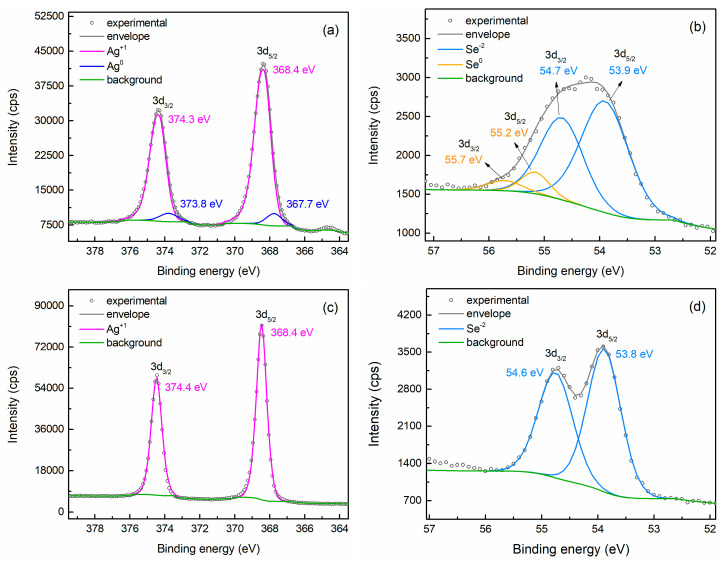
High resolution XPS spectra of Ag 3d and Se 3d orbitals acquired from samples PC3 (**a**,**b**) and PC4 (**c**,**d**). The fitting analysis reveals minor contributions from metallic Ag and Se in PC3, whereas in PC4 only the bonds corresponding to Ag_2_Se are detected.

The chemical state of silver and selenium in PC3 and PC4 was thoroughly investigated through XPS analysis, and the high-resolution spectra of Ag 3d and Se 3d orbitals for both samples are given in Figure 2. All profiles are fitted using doublets to account for the 3d_3/2_–3d_5/2_ spin–orbit splitting. For powder PC3, the Ag 3d spectrum can be fitted with two components (Figure 2a). The main component exhibits peaks at 368.4 eV and 374.3 eV for the 3d_5/2_ and 3d_3/2_ states, respectively, while a secondary doublet appears at slightly lower binding energies, i.e., 367.7 eV and 373.8 eV. The primary doublet is ascribed to the Ag(I) valence state in Ag_2_Se [33,34,35] and accounts for 92.7% of the silver bonds. The low-energy weaker peaks correspond to metallic Ag(0), confirming the presence of Ag clusters [36,37]. The Se 3d signal also consists of two components as well, with overlapping spin–orbit peaks (Figure 2b). The main Se 3d_5/2_–3d_3/2_ doublet, with binding energies of 53.9 eV and 54.7 eV and spin–orbit coupling of ~0.8 eV, can be assigned to the Se^2-^ oxidation state in Ag_2_Se [33,34,35], accounting for 90.1% of the selenium bonds. However, the high-energy sub-peaks, centered at 55.2 eV (3d_5/2_) and 55.7 eV (3d_3/2_), are attributed to Se(0), indicating the presence of a limited amount of elemental Se [38] that was not detected by the previous characterization techniques. XPS analysis therefore confirmed that small quantities of elemental Ag and Se remain after the three-hour synthesis. Conversely, the profiles of both Ag 3d and Se 3d spectra of sample PC4 can be fitted with a single component (Figure 2c,d). The Ag 3d_5/2_ peak at 368.4 eV is attributed to the Ag(I) valence state in Ag_2_Se, while the Se 3d_5/2_ peak with binding energy 53.8 eV originates from the Se^2-^ oxidation state in Ag_2_Se. The absence of secondary contributions in both Ag and Se bonds indeed suggests that the synthesis process reaches completion after 4 h of heating.

In previous studies, a deterioration in the thermoelectric performance of silver selenide in the presence of excess silver was reported [10,39,40,41]. Abrikosov proposed that most of the excess silver atoms form metallic clusters, which dope the material with extra electrons [42], resulting in higher carrier density and reduced Hall mobility. While the electrical conductivity is enhanced, the Seebeck coefficient is significantly suppressed, leading to decreased *ZT* values as the figure of merit is proportional to *σ* and *S^2^*. Considering these effects, the optimum Ag_2_Se powder synthesized in the current work is clearly sample PC4, in which no residual Ag and Se was detected.

**Figure 3:**
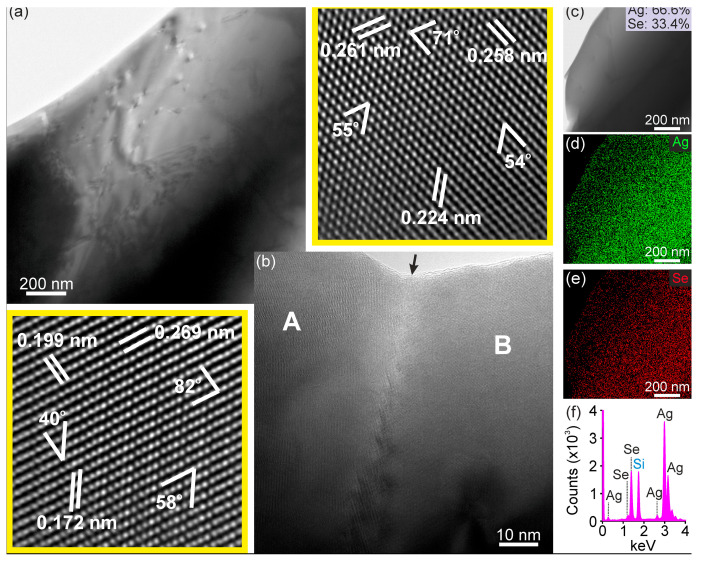
TEM micrograph of sample PC4 showing part of a large grain (**a**). HRTEM image of a bicrystal with Moiré fringes at the grain boundary (arrow) (**b**). Insets display atomic-resolution images of grains A (**left**) and B (**top**), with corresponding lattice *d*-spacings and angular measurements for crystallographic indexing. STEM image of a grain region used for compositional analysis confirming a stoichiometric Ag:Se ratio (**c**). EDS elemental maps demonstrating uniform spatial distribution of Ag and Se (**d**,**e**). Representative EDS spectrum from the mapped area (**f**). The X-ray peak associated with Si arises from sample preparation.

TEM methods were employed to investigate further the grain morphology, microstructural features, and phase composition of sample PC4. The results confirm that the Ag_2_Se powder adopts a polycrystalline character, consisting of randomly oriented grains with dimensions typically on the order of a few micrometers. These grains are interconnected through random grain boundaries, indicative of a heterogeneous but well-consolidated microstructure. TEM imaging reveals that the defect density within the crystallites is relatively low. Only a limited number of extended defects, such as isolated dislocations and occasional stacking faults, were observed, suggesting that the synthesis process yields microcrystals of comparatively high structural quality (Figure 3a). The predominance of defect-free regions within the grains is consistent with the good crystallinity expected for Ag_2_Se and supports the consistency of subsequent structure–property correlations.

Phase identification of the Ag_2_Se crystallites was performed by detailed high-resolution TEM (HRTEM) analysis. The procedure involved measurements of the lattice *d*-spacings from multiple crystallographic planes, along with angular relationships between intersecting planes, to establish unambiguous structural assignments and enhance the reliability of the analysis. Figure 3b presents an HRTEM image of an Ag_2_Se bicrystal, projected along a direction in which the grain boundary is inclined with respect to the electron beam. The overlap of the two crystallographically equivalent grains of different orientations across the boundary gives rise to characteristic Moiré fringes, which clearly delineate the interfacial region. High-magnification images of the atomic structure of each grain are provided as insets, together with the corresponding *d*-spacing and angular measurements obtained using the procedure described above. The corresponding results are shown in Table 1. The experimental measurements were systematically compared with reference data provided in the JCPDS/ICDD database (PDF cards). Thus, for grain A, the (112), ($1\bar{3}1$), and (041) lattice planes of the orthorhombic *β*-Ag_2_Se phase were unambiguously identified, while for grain B the identified planes correspond to ($00\bar{3}$), ($12\bar{1}$) and (122) planes, belonging to the same orthorhombic phase. From these assignments, the angle between the [$71\bar{4}$] and [$2\bar{1}0$] zone axes of crystals A and B, respectively, was determined to be ~64.4°, in excellent agreement with the theoretical value calculated from the orthorhombic lattice parameters *a* = 0.433 nm, *b* = 0.706 nm and *c* = 0.776 nm lattice constants. This confirms both the accuracy of the experimental indexing procedure and the crystallographic equivalence of the two adjoining grains, with the observed Moiré fringes arising from their relative misorientation rather than a phase transition across the boundary. Several other measurements in various crystals resulted in identification of the same phase, suggesting that the sample is composed exclusively of the *β*-Ag_2_Se phase.

Complementary EDS measurements were performed at various grains to evaluate the elemental distribution and compositional uniformity of the sample. As shown in Figure 3c,e, the EDS elemental maps confirm a homogeneous distribution of both Ag and Se across the grains, without evidence of Ag- or Se-rich areas. Quantitative results were obtained by using the Oxford Aztec 4.0 software. A representative spectrum is displayed in Figure 3f. The corresponding elemental analysis shown in Figure 3c demonstrates an Ag:Se atomic ratio very close to the stoichiometric *β*-Ag_2_Se phase. Importantly, consistent results were obtained across all probed areas, further confirming that the sample exhibits both structural and chemical homogeneity. These findings in conjunction with the HRTEM analysis demonstrate that the PC4 sample is composed exclusively of the *β*-Ag_2_Se phase, with chemical uniformity directly corroborating the structural identification.

**Figure 4:**
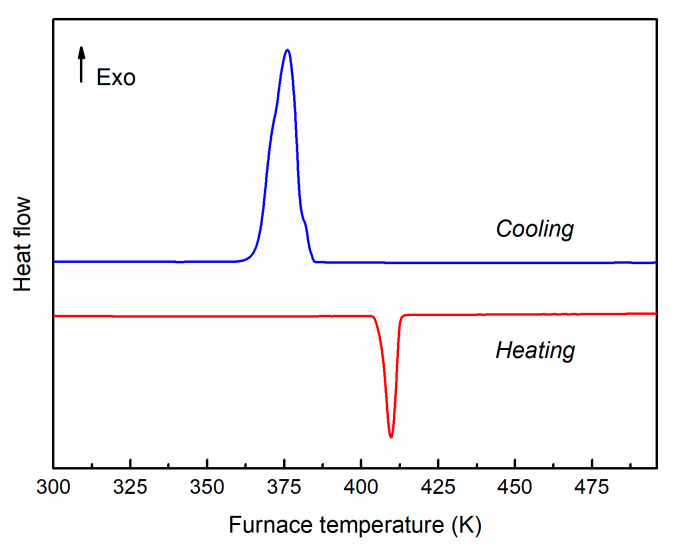
DSC heating and cooling curves of sample PC4, depicting the phase transition of Ag_2_Se from *β*- to *α*-phase.

Figure 4 shows the DSC heating and cooling curves of sample PC4. The prominent endothermic peak observed at about 409 K upon heating is attributed to the phase transition from orthorhombic *β*-Ag_2_Se to cubic *α*-Ag_2_Se, in good agreement with the literature [43,44,45]. During cooling, a corresponding exothermic peak appears, shifted to 376 K, reflecting the reversible nature of the transition. The hysteresis in the phase transition is an interesting feature of Ag_2_Se, as previously reported [44,45].

The Ag_2_Se powder was subsequently consolidated into a pellet to evaluate its thermoelectric performance. Consolidating Ag_2_Se is quite challenging; the cold press method results in poor mechanical strength, making the densification of Ag_2_Se through sintering processes like hot pressing or SPS inevitable. The sintering temperature is typically well-above 409 K, where the transition from orthorhombic to superionic cubic structure occurs. In the superionic phase, the Ag^+^ ions become highly mobile (e.g., with ion self-diffusion coefficient of 0.43 × 10^−5^ cm^2^ at 500 K [46]), leading to a less controlled distribution of silver. Potential Ag ion migration and Se volatilization during the densification process may cause elemental inhomogeneity, Ag precipitates at the grain boundaries, and off-stoichiometry in the sintered pellets, thereby affecting thermoelectric properties. Therefore, the compositional homogeneity and phase purity of the hot-pressed pellet PC4 were examined prior to thermoelectric measurements.

**Figure 5:**
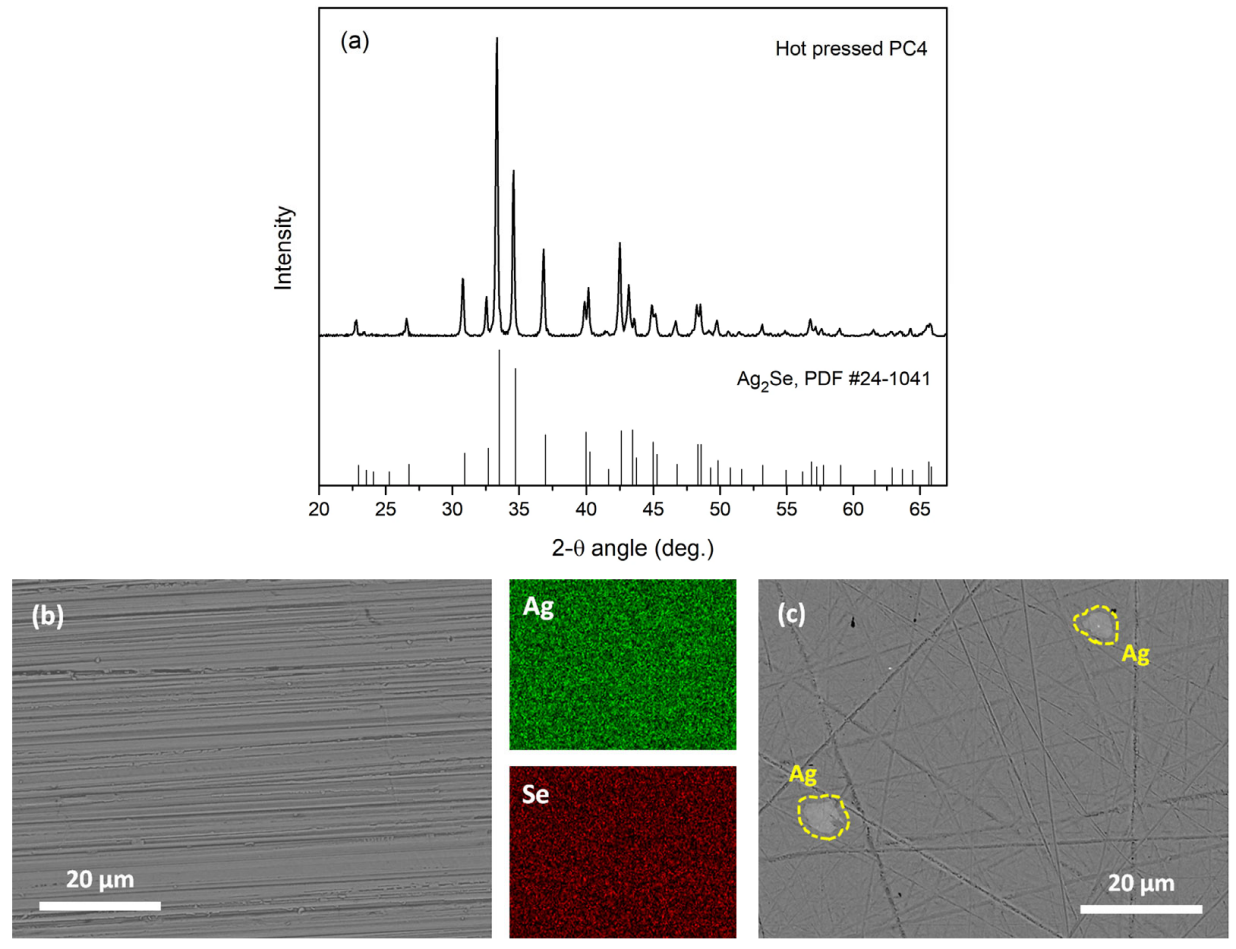
XRD pattern of pellet PC4 (**a**). Backscattered electron SEM image of pellet PC4 with the corresponding Ag and Se elemental maps (**b**). Backscattered electron SEM image of pellet PC3 depicting the formed Ag clusters (**c**).

The XRD pattern of the pellet is essentially the same as that of the powder, exhibiting only the peaks of orthorhombic *β*-Ag_2_Se (Figure 5a). Figure 5b shows a typical backscattered electron SEM image of the surface of pellet PC4, along with the corresponding EDS elemental maps. The pellet is dense, and no micropores—which would have a negative impact on the electrical transport properties—are observed. This contrasts with other industrial synthesis methods, such as mechanical alloying combined with SPS, which often induce considerably large porosity in Ag_2_Se and other thermoelectric materials [44,47,48,49]. Ag and Se are homogeneously distributed in PC4, and no secondary phases or Ag clusters are observed. For direct comparison, a backscattered electron image of pellet PC3 is also provided (Figure 5c), where the contrast is evident, with clearly visible Ag precipitates of several micrometers in size present on the surface. As already discussed, residual Ag impurities had been detected in the powder form of PC3, and Ag ion movement during the consolidation process may have further exacerbated this issue. The actual mean chemical composition of pellet PC4, determined by EDS analysis, is Ag_2.14_Se, which slightly deviates from the stoichiometric 2:1 ratio. Given that no apparent silver clusters or micropores are observed, it is speculated that this minor off-stoichiometry might arise from: (a) a limited number of mobile Ag^+^ ions occupying interstitial sites in the lattice during the superionic state, and (b) negligible Se volatilization due to the sintering process. Either of these reasons, or a combination of both, could potentially result in some Ag-rich regions on the nanometer scale that cannot be observed by SEM-EDS. Huang et al. [47] made a similar observation in their work, where three-dimensional atom probe tomography revealed Ag-rich and Se-rich nano-precipitates in the seemingly pure matrix, which were undetectable by EDS mapping and affected the transport properties.

**Figure 6:**
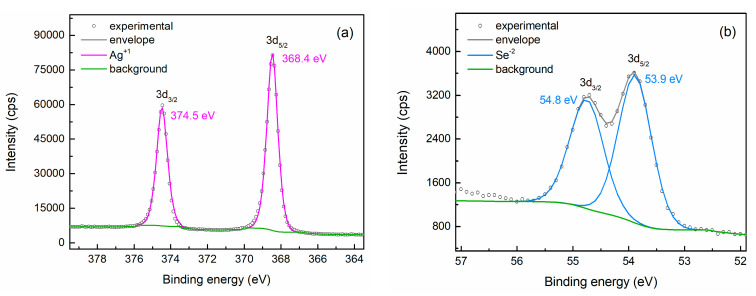
High resolution XPS spectra of Ag 3d (**a**) and Se 3d (**b**) orbitals acquired from pellet PC4, fitted with doublets corresponding to the bonds in Ag_2_Se.

The XPS high resolution Ag 3d and Se 3d spectra acquired from pellet PC4 (Figure 6) display the same components as those of the corresponding powder (Figure 2c,d). No evident contribution from Ag^0^ was revealed in the Ag 3d spectrum, as the profile is well-fitted with the doublet corresponding to the Ag^+1^ state (Ag 3d_5/2_ at 368.4 eV). Similarly, only the Se^−2^ oxidation state of Ag_2_Se is present in the Se 3d spectrum (Se 3d_5/2_ at 53.9 eV). It should be noted, however, that a negligible contribution from metallic Ag traces would probably be indistinguishable due to strong overlapping with the primary Ag^+1^ component. Combining these results with the findings from the SEM-EDS analysis strengthens the hypothesis that only limited movement of Ag ions and/or Se vacancies occurred during the consolidation of powder PC4, and the material was not considerably degraded; it remained relatively close to the stoichiometry of Ag_2_Se while possibly containing a small amount of scattered Ag-rich nanoregions. Nonetheless, since the thermoelectric performance of Ag_2_Se is highly sensitive to small variations in the Ag:Se ratio and defects [16,50,51], this information should be taken into consideration when interpreting the thermoelectric properties.

### 3.2. Thermoelectric Properties

The electrical transport properties of the synthesized Ag_2_Se material at room temperature were evaluated through Hall measurements. The carrier concentration was determined to be *n_H_* ≈ 5 × 10^19^ cm^−3^, the Hall coefficient *R_H_* = −0.12 cm^3^ C^−1^, and the corresponding mobility *μ_H_* = 203 cm^2^ V^−1^ s^−1^ at 300 K. The negative sign of the Hall coefficient is related to the *n*-type conductivity of the material, with electrons being the majority carriers. The theoretically optimum carrier concentration of Ag_2_Se, as predicted by single parabolic band model calculations, is about 1.6 × 10^18^ cm^−3^ at 300 K [20], which is interestingly one to two orders of magnitude lower than that of most thermoelectric materials. The relatively higher *n_H_* value obtained in this work can be attributed to the increased electron carriers associated with limited Ag ion drift and Se loss during sintering, which leads to non-stoichiometric defects and the slight silver excess detected by elemental analysis. The potential presence of Ag nano-precipitates could further contribute to the mediocre carrier mobility [13,47]. Previous studies, emphasizing the great impact of carrier concentration on the transport properties of Ag_2_Se, have predicted a rapid fall in carrier mobility when the carriers increase in order of magnitude above 10^18^ cm^−3^ [11,20,43]. Hence, the thermoelectric transport properties are not optimized yet. A commonly followed strategy to decrease its carrier density and improve mobility involves the addition of a small amount of Se to synthesize Se-rich Ag_2_Se (Ag_2_Se_1+δ_) [13,14,15,50]. Anion excess atoms act as acceptors in n-type Ag_2_Se; thus, even less than 1% of anion excess can drastically drop *n_H_* to significantly lower values. Moreover, Se excess was reported to suppress potential metastable phases and prevent the formation of Ag precipitates in Ag_2_Se-based materials [13,51]. A future experimental investigation of Se-rich Ag_2_Se synthesis using pack cementation could optimize the transport properties of the produced material.

**Figure 7:**
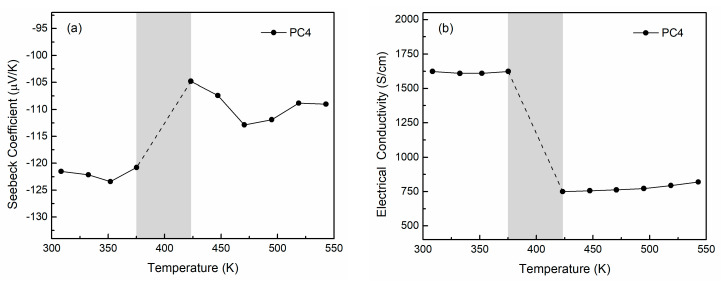

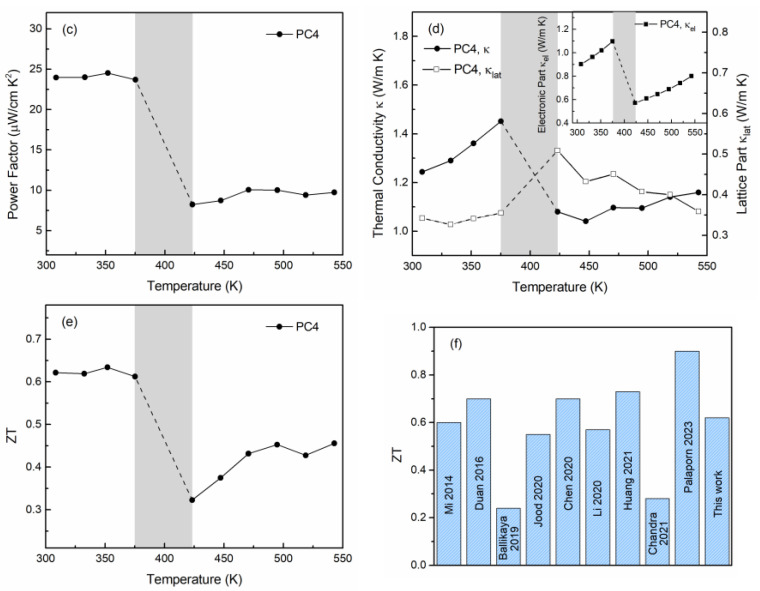
Thermoelectric performance of PC4 in terms of Seebeck coefficient (**a**), electrical conductivity (**b**), power factor (**c**), thermal conductivity (**d**), and figure of merit *ZT* (**e**), along with a comparative graph of reported *ZT* values (**f**). The data in the gray region between 375–423 K are omitted, as this area corresponds to the phase transitioning, which leads to anomalies in the measurements.

Figure 7 presents the thermoelectric properties of PC4 over the temperature range of about 310–545 K. An anomaly is recorded in the measured values of all properties between 375 K and 423 K, corresponding to the phase transformation region of Ag_2_Se, as verified by the DSC curves. This area is shown in gray in the property plots and is excluded from further discussion. Figure 7a demonstrates the Seebeck coefficient (*S*) of PC4. The negative values confirm the dominant n-type conduction, in agreement with the Hall measurements, and the overall trend of *S(T)* reflects the changes occurring due to the *β*- to *α*-phase transition. At low temperatures, *S* exhibits limited variation, with a value of −121.5 μV K^−1^ at 310 K. A decreasing tendency is observed for T ≥ 350 K as the material enters the bipolar region, consistent with the narrow bandgap of *β*-Ag_2_Se. The abrupt reduction in the $\left|S\right|$ value, from 120.8 μV K^−1^ at 375 K to 104.8 μV K^−1^ at 423 K, originates from the transformation of the orthorhombic to the cubic structure. The Seebeck coefficient is inversely proportional to the carrier concentration, according to Equation (1):

(1) $$S=\frac{8\pi^{2}k_{B}^{2}}{3eh^{2}}m^{*}T{\left(\frac{\pi }{3n}\right)}^{\frac{2}{3}},$$

where *h* is Planck’s constant, m* is the effective mass, and *k_B_* is the Boltzmann’s constant. In the region of phase transitioning, the Hall carrier concentration of Ag_2_Se is a dramatically increasing function of temperature, which explains the sharp drop that the absolute *S* value suffers after the conversion. Above 423 K, *S* again shows a slight increasing trend, with a value of −109 μV K^−1^ at about 545 K, although it remains significantly lower than in the *β*-phase region. The maximum absolute value of the Seebeck coefficient is $\left|S_{max}\right|=122.7\;\mu VK^{-1}$ at 352 K, which is close to values reported in previous studies, taking into account the relatively higher carrier concentration in sample PC4 [13,14,24,41]. The bandgap (*E*_g_) can be roughly estimated using the Goldsmid’s relation based on the maximum Seebeck coefficient and the corresponding temperature, $E_{g}=2eS_{max}T_{max}$ [52], giving a value of approximately 0.086 eV.

The variation of electrical conductivity (*σ*) with temperature is plotted in Figure 7b, and the curve’s shape follows prior reports on Ag_2_Se [14,19,41,43]. Consistent with the observed changes in the Seebeck coefficient, *σ* exhibits weak temperature dependence below 375 K, with a subtle increasing trend. Considering the opposite tendencies of the two quantities, it can be inferred that a small increase in the carrier concentration of PC4 occurs within this temperature range, further supporting its semiconducting nature. A high value of *σ* = 1623.7 S cm^−1^ is measured at 310 K, which is comparable to the values reported by Mi et al. [14], Lin et al. [43], and other researchers [47,53]. The electrical conductivity sharply drops from about 1624 S cm^−1^ at 375 K to 750 S cm^−1^ at 423 K, owing to the transition from semiconducting to metal-like behavior. As the material enters the superionic state, the Ag ions move freely and tend to scatter electrons more effectively than in their former static sites, leading to a drastic decrease in carrier mobility [19,20,54]. In the high-temperature region, *σ* displays a slight increasing trend with temperature, but its values are significantly lower than those measured for the low-temperature phase.

The electrical performance of PC4 is reflected in the calculated power factor, *PF = S^2^ σ* (Figure 7c). The *PF* value is significantly reduced after the phase transition, and it is clear that the electrical properties of orthorhombic *β*-Ag_2_Se are superior to those of cubic *α*-Ag_2_Se. By combining the high electrical conductivity with the reasonably large Seebeck coefficient, a maximum power factor of 24.5 μW cm^−1^ K^−2^ is obtained at 352 K, which is among the highest values reported for Ag_2_Se [8,11,14,24,47].

Figure 7d presents the temperature-dependent thermal conductivity (*κ*). The total thermal conductivity is *κ* = 1.24 W m^−1^ K^−1^ at 310 K, and is expected to peak as the material enters the phase transition region due to the substantial change in specific heat associated with the structural transformation. After the conversion, κ drops to 1.08 W m^−1^ K^−1^ at 423 K and then gradually rises, following a trend similar to that of σ and approaching the magnitude observed at low temperatures, as also reported by other researchers [8,43]. Since κ consists of the lattice (*κ*_lat_) and electronic (*κ*_el_) components, expressed as *κ* = *κ*_lat_ + *κ*_el_, its trend must be interpreted by considering both contributions. In the low-temperature region, κ gradually increases with temperature. This behavior contrasts with the decreasing trend typically observed when Umklapp phonon-phonon scattering dominates, indicating a major contribution from the electronic part to thermal transport. Day et al. [20] and Jood et al. [13] calculated *κ*_el_ of Ag_2_Se to represent ≥ 60% of *κ* below the phase-transition region, implying that the total thermal conductivity of this compound is more sensitive to changes related to charge carriers rather than to the phonon spectrum. In this study, the electronic part was estimated according to the Wiedemann-Franz law, *κ*_el_ = *L σ Τ*, where *L* is the Lorentz number (*L* ≈ 1.8 × 10^−8^ V^2^ K^−2^ for Ag_2_Se) [13], and the lattice contribution was obtained by subtraction. Both components are presented in Figure 7d. The *κ*_el_ indeed accounts for over 56% of the total thermal conductivity throughout the temperature range, with a maximum of 76% at 375 K, where the phase transition begins. Thus, the increasing trend of *κ* before and after the phase change mainly arises from the electronic part, underscoring the importance of tuning the carrier concentration in the material. Conversely, the lattice contribution shows negligible variation at low temperatures and decreases after the conversion. Its values are particularly low, ranging from 0.33 to 0.51 W m^−1^ K^−1^, consistent with a disordered crystal possessing inherently low κ_lat_. The moderately low total thermal conductivity of the prepared material falls within the range reported elsewhere for low temperatures [12,13,14,20,53]. These values could be linked to the relatively higher carrier concentration found in this work, pointing to the presence of a limited Ag excess at interstitial sites in the PC4 matrix, potentially forming nanoregions that demonstrate higher thermal conductivity.

The overall thermoelectric performance of the Ag_2_Se sample is evaluated through the calculated figure of merit (Figure 7e). The high power factor and the relatively low thermal conductivity yield a peak *ZT* of 0.63 at 352 K. Small deviations around this value are observed in the range 310–375 K, followed by a pronounced decline due to the phase transition to the cubic phase. This indicates that the prepared material maintains a quite stable performance up to about 375 K, suggesting potential operation over a broad low-temperature window with minimal efficiency variations. Although the maximum *ZT* does not reach the extraordinary values above 0.8 reported by Chen et al. [11] and Palaporn et al. [53] below 400 K, it is still within the respectably good range of *ZT*_max_ = 0.5–0.75 achieved in previous studies for pristine Ag_2_Se [12,13,14,21,24,47], thereby competing with conventional and alternative synthesis routes proposed elsewhere. Figure 7f shows a comparative chart of room-temperature *ZT* values for Ag_2_Se published in the literature along with the one reported in the present work [11,12,13,14,24,47,53,55,56]. As already discussed, the increased carrier concentration has a significant impact on the figure of merit of Ag_2_Se, affecting both its electrical and thermal transport properties [11,20]. Tuning the carrier concentration to the low 10^18^ cm^−3^ range –for instance, through Se excess or doping with S—could push the *ZT* even beyond 1 [8,13], which is of practical interest and renders the material competitive with commercial low-temperature Bi_2_Te_3_ materials. Future studies should further explore the prospects of the pack cementation technique in this direction, aiming to establish it as a viable route for producing optimized high-*ZT* Ag_2_Se materials.

## 4. Conclusions

Ag_2_Se powder was successfully synthesized through the pack cementation technique, identified as *β*-Ag_2_Se with an orthorhombic structure and free of secondary phases. The elemental composition was close to the stoichiometric 2:1 ratio, and the grain size was typically on the order of a few micrometers. Consolidation by hot pressing did not induce substantial changes in the material, which remained *β*-Ag_2_Se with no Ag or Se micro-precipitates and no pores observed. A slight silver excess detected in the matrix (Ag_2.14_Se) can be attributed to limited Se volatilization and Ag^+^ ion migration to interstitial sites due to heating at elevated temperatures above the superionic transition. This process may also have led to the formation of Ag-rich nanoregions, undetectable on the micro-scale. The above explanation is supported by the high carrier concentration (~10^19^ cm^−3^) measured, which reduces the carrier mobility to mediocre values. The combination of sufficiently high electrical conductivity and a reasonably large Seebeck coefficient yielded the high power factor of *PF_max_* = 24.5 μW cm^−1^ K^−2^ at 352 K. The thermal conductivity was strongly dependent on the charge carrier density, leading to moderately low values (*κ* = 1.24 W m^−1^ K^−1^ at 310 K), again suggesting the possible presence of Ag nano-precipitates with intrinsically high *κ*. As a result, Ag_2_Se achieved a peak figure of merit of *ZT_max_* = 0.63 at 352 K. The respectably good thermoelectric performance of pristine Ag_2_Se synthesized by pack cementation can match or even surpass results obtained through conventional or other proposed routes. Tuning the carrier concentration to lower values through anion excess could further enhance *ZT* to levels of practical interest. This work has laid the foundation for further exploration of pack cementation as a promising pathway for the Ag_2_Se synthesis, with the prospect of enabling cost-effective mass production of optimized silver selenide materials for low-temperature thermoelectric applications.

## Figures and Tables

**Table 1:** HRTEM experimental measurements of *d*-spacing values and angles between intersecting crystal planes in synthesized Ag_2_Se.

*d*-Spacing Grain A (nm)	Angle (°)	*d*-Spacing Grain B (nm)	Angle (°)
*d*_1_ = 0.269 nm	<*d*_1_*d*_2_> = 82	*d*_1_ = 0.261 nm	<*d*_1_*d*_2_> = 71
*d*_2_ = 0.199 nm	<*d*_2_*d*_3_> = 40	*d*_2_ = 0.258 nm	<*d*_2_*d*_3_> = 54
*d*_3_ = 0.172 nm	<*d*_3_*d*_1_> = 58	*d*_3_ = 0.224 nm	<*d*_3_*d*_1_> = 55

## Data Availability

The datasets presented in this article are not readily available because the data are part of an ongoing study. Requests to access the datasets should be directed to the corresponding authors.
